# The Libby Method

**Published:** 1897-11

**Authors:** J. L. Wolf

**Affiliations:** Washington, D. C.


### The Libby Method.

BY DR. J. L. WOLF, WASHINGTON, D. C.

Abstract of paper read at Southern Dental Association, Old Point Comfort, Va. August, 1897.

Having had during more than eighteen months' experience, opportunities for observing the most gratifying results from the use of the Libby hand-pressure gold pluggers, supplemented by the Rogers mallet points, especially in those cases which, by virtue of unusual difficulty attending the insertion of the filling, furnished excellent tests as to the efficiency of the instruments, it seems of sufficient importance, and this is a fitting occasion, to call the attention of those who may not be entirely satisfied with the results obtained by the generally accepted methods. The feeling of confidence as to the results which follow the use of this method contributes greatly to avert much of the sensation of exhaustion often experienced.

As mallet points supplementing the Libby hand-pluggers, the Rogers points are of incalculable value for the two-fold purpose of conservation of physical strength and condensation of material. With the Russel electro-magnetic mallet as the propulsive power they are capable of a very wide range of application.

